# Wellness through a comprehensive Yogic breathing program – A controlled pilot trial

**DOI:** 10.1186/1472-6882-7-43

**Published:** 2007-12-19

**Authors:** Anette Kjellgren, Sven Å Bood, Kajsa Axelsson, Torsten Norlander, Fahri Saatcioglu

**Affiliations:** 1 Department of Psychology, Karlstad University, Karlstad, Sweden; 2 Department of Molecular Biosciences, University of Oslo, Oslo, Norway

## Abstract

**Background:**

Increasing rates of psychosocial disturbances give rise to increased risks and vulnerability for a wide variety of stress-related chronic pain and other illnesses. Relaxation exercises aim at reducing stress and thereby help prevent these unwanted outcomes. One of the widely used relaxation practices is yoga and yogic breathing exercises. One specific form of these exercises is Sudarshan Kriya and related practices (SK&P) which are understood to have favourable effects on the mind-body system. The goal of this pilot study was to design a protocol that can investigate whether SK&P can lead to increased feeling of wellness in healthy volunteers.

**Methods:**

Participants were recruited in a small university city in Sweden and were instructed in a 6-day intensive program of SK&P which they practiced daily for six weeks. The control group was instructed to relax in an armchair each day during the same period. Subjects included a total of 103 adults, 55 in the intervention (SK&P) group and 48 in the control group. Various instruments were administered before and after the intervention. Hospital Anxiety Depression Scale measured the degree of anxiety and depression, Life Orientation Test measured dispositional optimism, Stress and Energy Test measured individual's energy and stress experiences. Experienced Deviation from Normal State measured the experience of altered state of consciousness.

**Results:**

There were no safety issues. Compliance was high (only 1 dropout in the SK&P group, and 5 in the control group). Outcome measures appeared to be appropriate for assessing the differences between the groups. Subjective reports generally correlated with the findings from the instruments. The data suggest that participants in the SK&P group, but not the control group, lowered their degree of anxiety, depression and stress, and also increased their degree of optimism (ANOVA; p < 0.001). The participants in the yoga group experienced the practices as a positive event that induced beneficial effects.

**Conclusion:**

These data indicate that the experimental protocol that is developed here is safe, compliance level is good, and a full scale trial is feasible. The data obtained suggest that adult participants may improve their wellness by learning and applying a program based on yoga and yogic breathing exercises; this can be conclusively assessed in a large-scale trial.

**Trial Registration:**

Australian Clinical Trial Registry ACTRN012607000175471.

## Background

Relaxation exercises offer a means to reduce the physiological and psychological reactions to stress [1,2]. Current achievement-based, demanding and high-tempo society has incurred increased risks and vulnerability for stress-related chronic pain and other illnesses [3-5]. A multitude of techniques for relaxation and stress-reduction are described, e.g. flotation-REST [6], Tai Chi Chuan [2], meditation [7] and yoga [8,9].

The different relaxation techniques often lead to specific psychological and physiological changes termed the 'relaxation response' (RR) [10]. The RR is identified as the physiological opposite of the stress or 'fight-or-flight response' [11]. The RR is associated with instantly occurring physiological changes that include reduced sympathetic nervous system activity, reduced metabolism, lowered heart rate, reduced blood pressure, and decreased respiratory rate [12,1]. At the psychological level, individuals typically report that RR techniques result in genuine rest, recovery from fatigue, better sleep quality, as well as an increased sense of control and efficacy in stressful situations [13].

Yoga is one of the many different techniques for achieving relaxation. Yoga has its origin in ancient India and in its original form consisted of a system of spiritual, moral and physical practices [9]. The most central and common aspects of yoga practice today are different bodily postures (asanas) and breathing exercises (pranayamas) [14,9] that aim to focus the mind, achieve relaxation and increase wellness. Various health benefits of yoga have been described in previous studies. A review of anti-depressive effects of different forms of yoga [9] indicated potential beneficial effects of yoga on depressive disorders. Other studies reported beneficial effects of yoga on anxiety, stress reduction and general well-being [15-17]. However, the results need to be interpreted carefully since many of the published studies about yoga are small and no systematic and comprehensive reviews of scientific research on yoga have been published. It may also be difficult to compare studies done on different forms of yoga since benefits of yoga practice might differ by the style of the practice [18].

Sudarshan Kriya and related Practices (SK&P) is a form of yoga practice that emphasizes breathing exercises. In addition to asanas, three different forms of pranayamas are practiced in succession [19]. Previous studies suggested that SK&P may be useful for relieving depression, improving the antioxidant defenses of the body, giving rise to beneficial EEG patterns, and possible improvements in blood chemistry [19-21]. For example, Janakiramaiah et al. found that the degree of depression significantly decreased (68–73 %) in subjects with clinical depression after they practised SK daily for three weeks and this decrease was as effective as conventional pharmacological treatment [19]. Another study indicated concurrent high activity of both alpha and beta waves in the EEG in SK practitioners indicating focus and relaxation at the same time, suggesting improved brain function [8]. A drop in blood lactate level, yet increase in the antioxidant enzymes superoxide dismutase, catalase, and glutathione, indicated favourable effects on antioxidant status [21].

The aim of the present study was to develop a protocol that can investigate whether SK&P could be considered in connection with increased wellness. Even though what constitutes wellness can be debated, there are a number of studies demonstrating that the variables included in this study are among the most relevant to feeling of wellness by different approaches [22-24]. To that end, outcome measures that were evaluated included depression, anxiety, mood, optimism, energy levels, and experience of altered states of consciousness.

## Methods

### Participants

This study included 103 participants (109 subjects to start with where 5 persons from the control and 1 person from the yoga group dropped out). The SK&P group was recruited through advertisement in the local paper and fliers. The control group was yoga naive, but had an interest in yoga and related practices, and were recruited in a similar way. The control group consisted of 55 participants (12 men, 43 females) and the yoga group of 48 participants (13 men, 35 females); mean age for the whole study was 31.92 years (SD = 11.33) (for the control group M = 31.90 years, SD = 10.84, and for experimental group M = 31.95 years, SD = 11.85).

The yoga group participated in the beginner's course in the SK&P program in November – December 2004 at the Art of Living Yoga Center in Karlstad and Eskilstuna, Sweden. The participants of the control group were recruited among yoga-interested students at Karlstad University. There were no significant differences between the groups (Mann-Whitney U test) regarding gender, occupation, education, phase in the menstruation cycle and usage of contraceptives (*ps *> 0.42). Analysis with Independent Samples T-test did not reveal any significant differences between the groups at baseline regarding age, degree of depression, stress, energy, ongoing pain, affective personality (PANAS-test), number of sleeping hours, minutes awake before falling asleep, experienced sleep quality, consumption of nicotine (cigarettes/snuff) or alcohol (*ps *> 0.11).

Comparison of the two groups at pre-test, before the intervention, indicated that there was a significant difference between the groups regarding degree of optimism and anxiety assessed by the Independent Samples T-test. The control group had lower degree of anxiety (*M *= 4.63, *SD *= 2.59) than the yoga group (*M *= 7.58, *SD *= 3.58) (*p *< 0.001, *t *_(101) _= 4.73). The control group also experienced higher degree of optimism (*M *= 24.88, *SD *= 3.86) compared to the yoga group (*M *= 21.84, *SD *= 5.72) (*p *= 0.002, *t *_(101) _= 3.11). The basis for these differences is not clear; however, the higher anxiety for the yoga group are not indicative of clinical anxiety diagnosis, and the values for optimism are in the normal range for both groups. Please see Table 1 for a comparison of baseline characteristics.

**Table 1:** Baseline characteristics:

	**SK&P**	**Controls**
**Age**	31.95 (11.85)	31.90 (10.84)
**Depression**	4.07 (3.078)	3.25 (2.026)
**Stress**	2.68 (0.93)	2.61 (0.92)
**Energy**	2.99 (0.91)	2.97 (0.77)
**Sleep Hours**	7.46 (0.84)	7.68 (0.50)
**Min Awake**	20.12 (18.79)	17.71 (13.27)
**Sleep Quality**	64.82 (21.37)	70.28 (17.94)
**cig/month**	12.47 (41.61)	15.00 (55.46)
**snuff/month**	1.40 (7,48)	0.13 (0.53)
**Alc ml/month**	184.915 (173.83)	156.73 (143.47)
**Optimism**	21.84 (5.72)	24.88 (3.86)
**Anxiety**	7.58 (3.58)	4.63 (2.59)

The measures were as described in Methods. 'Sleep Hours' refers to number of daily sleep, 'Min Awake' the number of minutes before falling asleep, 'Sleep Quality' refers to perceived quality of sleep, 'cig' are number of cigarettes per month, 'Alc' is alcohol. The numbers in parentheses are standard deviation.

Several criteria were used for subject inclusion and exclusion. Inclusion: having an interest in yoga and relaxation exercises and that they would like to practice some kind of relaxation practise daily for 6 weeks. Exclusion: pregnancy, ongoing psychiatric disease, younger than 18 years, clinical depression or anxiety-problems.

The study was approved by the Ethical Committee at Karlstad University. All participants signed consent forms and they were treated according to the ethical guidelines of APA. Participants were informed that their data will be kept anonymous, participated by their free will and were free to leave the experiment whenever they wished without giving any reason for it.

### Description of intervention

Sudarshan Kriya (SK) and related practices (SK&P) are derived from the Yogic Science of Breath derived from Vedic texts. SK&P includes gentle stretches (yoga postures), specific breathing exercises (the central technique is SK; see below), and cognitive coping and stressor evaluation strategies. SK&P is traditionally understood to dissolve emotional distress and create the subjective experience of rest and well-being. The instructors in SK&P were trained by the International Art of Living Foundation in two residential courses of two weeks each which included approximately one year of practical field work in between.

The breathing techniques that are part of SK&P are: (a) Three-Stage Pranayama with Ujjayi or "Victory Breath", (b) three sets of Bhastrika or "Bellow's Breath", and (c) SK or the "Healing Breath Technique" and they were practiced in that order. The breathing practices are done in a sitting posture, either in a chair or on the floor. Eyes are kept closed throughout the sessions.

Normal breathing is at the rate of 14 to 16 breaths per minute. Ujjayi is a slow and deep breathing technique at 2 to 4 breaths per minute. Three-Stage Pranayama with Ujjayi breath is an advanced form using a specific ratio of inhalation and exhalation, and breath-holds. Participants practice this component where specific arm positions are held for approximately ten minutes in total.

The second breathing component of SK&P is Bhastrika. Here the breathing is vigorous and faster, about twenty to thirty respiratory cycles per minute. Three approximately one-minute rounds of Bhastrika are followed by a few minutes of normal breathing. Arm movements are used to increase the force and depth of inhalation and exhalation. Practice of this component lasts for approximately five minutes.

The central component of SK&P is SK which is an advanced cyclical breathing exercise of slow, medium, and fast rates in succession. Slow breaths are about 20 respiratory cycles per minute, medium breaths are about 40–50 respiratory cycles per minute, and the fast breathing is about 60–80 cycles per minute. The participant rotates through these breathing patterns during SK. Daily home practice of SK takes approximately 10 minutes. During the instruction phase, several longer group sessions of SK, lasting approximately thirty minutes, are practiced.

SK&P course was held in 6 consecutive days of approximately 3 hours each. Ujjayi breath and bhastrika were introduced on the first day, yoga on day 2, and SK on days 3 and 4. All techniques were performed everyday from day 3. Discussion of different cognitive coping and stressor evaluation strategies took place at different time points throughout the course.

The control intervention consisted of sitting in an armchair with the eyes closed and with gentle attention on the breath, and lasted for at least 15 min. per session. If thoughts were coming, participants were instructed to let go of the thoughts and start counting their breathing from 1 to 10 (Benson-breathing) [10].

### Design

The current study used a two-way split plot design. *Time *was relaxation (sitting in an armchair, or yoga) with assessments before and after the treatment period (6 weeks) and it constituted the within-subject factor and where *Treatment *(control, yoga) constituted the between-subject factor. The treatment for the yoga group was a six-day introduction course in SK&P and thereafter daily home practice (approximately 1 hour) for the next 5 weeks. The control group participants had meetings with the experimenter during the first six days for the same amount of time as the SK&P group. They met in a silent room and were instructed to relax in an armchair for 15 minutes; after that, they performed armchair relaxation daily during the remaining 5 weeks.

The dependent variables measured were: depression, anxiety, dispositional optimism, stress, energy and degree of altered states of consciousness. Qualitative data (written reports) about personal experiences were collected twice during the treatment period.

### Procedure

In the very beginning, the participants were informed that their participation is voluntary and that they can leave the study at anytime without the need for any explanation. The participants then answered questionnaires for background data, as well as the tests HAD, LOT, SE, PANAS and PAI (see instruments) in random order. The yoga group then began their SK&P course of six days. The control group also met with a course leader for six consecutive days in a dimly lighted room and relaxed in an armchair for 15 min each session. At day 4, the EDN test (see Instruments) was given and in the end of day 6 verbal written reports on experiences of the interventions were collected as qualitative data.

A final assessment was done at the end of the treatment period (6 weeks) with the test instruments HAD, LOT, and SE in randomized order. A second qualitative written report was collected in response to the question "Have these six weeks resulted in any changes in your life?" as well as checking how many times per week they have performed the relaxation exercises. The extent of home practice during the treatment period was monitored by a written self-report in response to the question 'How many days of the week did you practice at home?' at the post-test session. Only those who practiced at least 3 times a week were included in the analysis.

### Instruments

#### Background data

Questions were included about participants' age, gender, educational level, occupation, usage of contraceptive pills and phase in menstruation cycle (for females), sleeping habits and consumption of tobacco and alcohol.

#### HAD – Hospital Anxiety Depression Scale

The HAD is a rating scale concerning degree of anxiety and depression. The HAD scale measures the degree of anxiety and depression, wherein values under 6 are considered normal, those between 6 and 9 are borderline and values over 10 points are indicative of a probable depression or anxiety diagnosis. It was originally constructed by Zigmond and Snaith [25], for use with physically ill people. It has since then been revised to be used as a rating scale for anxiety and depression. The instrument consists of fourteen statements with four response alternatives (i. e. 0 to 3), ranging from positive to negative or vice versa and there are seven statements regarding anxiety and seven regarding depression. The validity and reliability of HAD has been described (Hermann, 1997 #32).

#### LOT – Life Orientation Test

The LOT test [26] consists of eight items, plus four filler items. The task of each subject is to take up a standpoint to the extent of whether or not one is in agreement with each of the items described, on a scale of 0 – 4, where 0 indicates "strongly disagree" and 4 indicates "strongly agree". The test measures dispositional optimism, defined in terms of generalized outcome expectancies. Parallel Test Reliability of LOT is reported as 0.76 and internal consistency was 0.76 (Scheier, 1985 #23). LOT is also regarded to have an adequate level of convergent and discriminate validity (Scheier, 1985 #23).

#### SE – Stress and Energy

The SE test is a self-estimation instrument concerning individuals' energy and stress experiences [27]. It consists of two subscales that elucidate the mood levels of the subjects on the dimensions: 'experienced stress' and 'experienced energy'. The response alternatives were arranged on six-grade scales, extending from: 0 = not at all, to 5 = very much. The instrument has been validated by analyses from studies focused on occupational burdens and pressures [28,29,27]. The SE-scale was constructed and based on an early and much used checklist, the Mood Adjective Check-List, constructed by Nowlis and Green [30] and modified further and translated into Swedish by Kjellberg and Bohlin [29]. Kjellberg and Iwanowski [27] reduced the list to 12 adjectives on two dimensions. It is currently the latest version of the SE-scale (with test-retest scores of 0.73 to 0.78) and was used in the present study. The test did not have a time limit.

#### PANAS – Positive affect and Negative Affect Scales

The PANAS-instrument [31-34] assesses the degree of affect, both negative (NA) and positive (PA). The instrument consists of 10 adjectives for the NA-dimension and 10 adjectives for the PA-dimension. In the test manual [34], it is postulated that the adjectives describe feelings and mood. The participants were asked to estimate how they had been feeling during the last week. Response alternatives are presented on 5-degree scales ranging from 0 = "not at all" to 5 = "very much". The PANAS-scale has been validated through studies focused upon several different routinely used scales within psychopathology [35].

#### PAI – Pain Area Inventory

The test, developed by Human Performance Laboratory, Karlstad University, for use in research studies on pain [36] and consists of two anatomical images of a human being, one frontal and one dorsal. The task of the participants is to indicate with a colour pen their areas of pain and colour them in. A transparent, plastic film is then placed over the colored areas on both figures and the numbers of colored areas are counted.

#### EDN – Experienced deviation from normal state

An instrument modified for use with floatation-REST [37,6] utilizing the internationally-applied psychometric instruments APZ-questionnaire and OAVAV [38] for obtaining judgments of altered states of consciousness and the relaxation response. Several studies indicate strong connections between altered states of consciousness and different RR techniques such as Qigong [39], Tai Chi [40] and muscle relaxation training [41]. In total, the EDN consists of 29 questions whereby each is responded to on a visual analogy scale (0–100). A complete "index of experience" was constructed from the points obtained from all 29 questions and were averaged to provide a "sum of experience." These values reflect the total experience of deviation from normal states. Cronbach's alpha for EDN was 0.973 in the present study. Typical EDN-values after an individual's experience of flotation-REST is around 30 EDN-points for the first experience of flotation-REST and 40 EDN-points for further occasions [42]. This should be compared to the experience of resting on a bed in a dark quiet room (15 points) [43].

### Data analysis

All statistical analysis was done using SPSS v13.1. The qualitative part was analysed by thematic analysis [44]. Statistical analysis were carried out using two-way split plot ANOVA with Time (before, after) as the within subject factor and Group (control, yoga) as between subject factor, and with depression, anxiety, optimism, stress and energy as the dependent variables.

## Results

### Personality variables

#### Depression

The analysis yielded a significant difference for Time [*F *_(1,91) _= 13.47, *p *< 0.001, *Eta*^2 ^= 0.13, *power *= 0.95] where the degree of depression diminished from 3.67 (*SD *= 2.56) to 2.97 (*SD *= 2.02) during the treatment period. There was a significant interaction between Time and Group [*F *_(1,91) _= 11.24, *p *= 0.001, *Eta*^2 ^= 0.11, *power *= 0.91] where the yoga group significantly lowered the degree of depression from 4.11 (*SD *= 2.99) at pretreatment to 2,73 (*SD *= 2.19) at posttreatment, while control group had a score of 3.25 (*SD *= 2.03) at pre-treatment and had no significant alteration at posttreatment (*M *= 3.19, *SD *= 1.84).

#### Anxiety

The analysis yielded a significant difference for Time [*F*_(1,91) _= 30.21, *p *< 0.001, *Eta*^2 ^= 0.25, *power *= 1.00] where the degree of anxiety diminished from 6.06 (*SD *= 3.50) at pretreatment to 5.19 (*SD *= 3.01) at posttreatment. Furthermore, there was a significant interaction between Time and Group [*F*_(1,91) _= 26.15, *p *< 0.001, *Eta*^2 ^= 0.22, *power *= 1.00] where the degree of anxiety in the yoga group significantly decreased, from 7.60 (*SD *= 3.71) at pretreatment to 5.87 (*SD *= 3.18) at posttreatment, while the control group did not show any significant alterations (from 4.63, *SD *= 2.59 to 4.56, *SD *= 2.72).

#### Optimism

The analysis yielded a significant difference for Time [*F*_(1,91) _= 4.55, *p *= 0.036, *Eta*^2 ^= 0.05, *power *= 0.56] where optimism increased from 23.14 (*SD *= 4.91) at pretreatment to 23.65 (*SD *= 4.62) at posttreatment. Furthermore, there was a significant interaction between Time and Group [*F*_(1,91) _= 9.86, *p *= 0.002, *Eta*^2 ^= 0.10, *power *= 0.87] where the degree of optimism was significantly increased in the yoga group from 21,29 (*SD *= 5.27) at pretest to 22.60 (*SD *= 5.16) at posttest, while the control group started at 24.88 (*SD *= 3.86) and had no significant alteration upon intervention (*M *= 24.63, *SD *= 3.84).

#### Stress

The analysis yielded a significant difference for Time [*F*_(1,89) _= 8.25, *p *= 0.005, *eta*^2 ^= 0.09, *power *= 0.81] where degree of stress diminished from 2.64 (*SD *= 0.92) to 2.41 (*SD *= 0.89) during the treatment period. There was also a significant interaction between Time and Group [*F*_(1,89) _= 9.06, *p *= 0.003, *Eta*^2 ^= 0.09, *power *= 0.85] where a significant decrease in stress for the yoga group was found where stress at pretest was 2.70 (*SD *= 0.92) and at posttest was 2.22 (*SD *= 0.85). For the control group there were no significant alterations during the treatment period (from 2.59, *SD *= 0.92 to 2.61, *SD *= 0.89).

#### Energy

The analysis yielded no significant differences or interactions regarding energy for either group (*ps *> 0.96).

#### Experience of altered states of consciousness – EDN scale

A one-way ANOVA was used to compare the means of EDN measurements. The analysis showed a significant difference between the control and yoga groups regarding EDN [F_(1,97) _= 177.14, p < 0.001, Eta^2 ^= 0.65, power = 1.00]. For the yoga group the EDN score of 42.50 (SD = 19.40) was significantly higher than that found for the control group of 3.19 (SD = 2.89).

## Discussion

The aim of this pilot study was to develop a protocol that can investigate whether SK&P can be of interest in connection with increased wellness in adult participants. As variables for wellness, a number of parameters were tested, such as degree of depression, anxiety, optimism, stress and energy.

The results suggested that the current protocol can be adopted for a full-scale trial and that the aspects of wellness measured in our study increased in the yoga group. The participants in the SK&P program had decreased level of depression and anxiety as well as increased degree of experienced optimism. There was also a decrease of stress experience in the SK&P group compared with the control group. Furthermore, SK&P induced an altered state of consciousness (ASC) during the practice of the program, but not in the control group.

The yoga group which practiced SK&P for 6 weeks decreased their degree of depression by 33% (according to the HAD scale) whereas the control group did not have any significant change during the treatment period. Earlier studies have shown that those with clinical depression can benefit from SK&P [19]. The current study, where only apparently healthy people without clinical depression were included, suggests that SK&P does not only have utility as a possible therapeutic strategy for patients, but also may be a method for healthy people who would like to increase their wellness.

The strong decrease in the experienced anxiety that the SK&P practitioners exhibited is interesting. There was no clinical anxiety diagnosis in the participants, but the yoga group had higher degree of anxiety before treatment (in the borderline zone between normal and anxiety diagnosis according to the HAD scale) compared with the control group. The SK&P group decreased the anxiety scores down to normal values after 6 weeks of practice.

Heightened optimism is another important aspect of the wellness experience. There was an increase in the degree of optimism in the SK&P group during the treatment period, whereas there were no changes in the control group. There was a difference in optimism before the treatment where SK&P group was less optimistic than the control group; the basis of this is currently unclear.

A reduction in stress was observed in the yoga group compared with no change in the control group. The value of a simple, functional method for stress reduction may be of significance. To have access to an effective method for stress reduction (i.e. SK&P) can lead to better health and a more effective daily life [7,10].

The increased experience of altered states of consciousness (ASC) during SK&P is also very interesting. Experience of ASC has been associated with deep relaxation and stress release [37,6]. That an ASC-state is achieved during SK&P can be interpreted as an indication of deep rest and relaxation. During an ASC-state many different psychological changes occur compared with normal waking state, for example, very deep relaxation, the feeling that the border between body and surroundings is eliminated, and this changes the sensation of time or mindset where new and creative thoughts are generated. A mild ASC-state is often described as having the character of daydreaming. This aspect of relaxation has previously been documented by our earlier work in connection with relaxation training in flotation-tank [6,42]. This technique is a method for deep relaxation where the subjects lie down on their back floating in a dark, isolated specially constructed tank filled with salty water at body temperature. The research on floatation tank therapy showed that the ASC-aspect of relaxation is an important aspect for inducing deep relaxation and other positive effects on quality of life [6,42]. Since SK&P was comparable to flotation rest in inducing ASC, it indicates that a very deep relaxation was experienced as a result of SK&P which could be the basis for the effects that are observed on wellness parameters.

The verbal descriptions from the subjects complement the quantitative data, for example in regards to reduced stress and a more optimistic outlook on life (data not shown). Here comes even other aspects of increased wellness as feeling of peace and balance, calming down, living in the present moment, and experience of a new outlook on life. The subjects who practiced SK&P felt that they have gained a tool that can be used in stressful situations that would make it easier to relax and better handle them. Furthermore, they experienced that yoga program decreased tensions, unpleasant sensations, blocks and locked-up feelings, as well as the experience of better control over their feelings. SK&P group expressed that it is easier to feel joy after they have learned this program. In addition, SK&P group had the sense that they had more energy and that the life force flowed through their body during the practice of this program. Despite their written statements on their experience of having more energy, this was not detected by statistical analysis based on the SE instrument. One possible reason for this could be that the increased energy that they have experienced was used to assimilate all the new impressions that they have received. These new impressions consisted, among others, increased thought activity about how they live their lives, how they think, and how they feel. Furthermore, it could be that the energy was used to handle the feelings and emotions that they previously suppressed. Additional work that utilizes multiple instruments is needed to assess these possibilities.

The shortcomings of this pilot study are that, first, this was not a randomised controlled trial. It is therefore possible that there has been a selection bias in the formation of the two groups. This could be alleviated with proper randomization in a full-scale trial. Second, checking for longer term effects of the interventions are desirable. Are they only effective for 6 weeks, or are they durable, as long as the techniques are practiced on a regular basis? This is important to determine for the possible long term utility of SK&P. Third, even though the two groups were treated as equally as possible, significantly more interaction occurred with the SK&P group and the instructor than that with the control group and its instructor which may have had some effects. Therefore, in a full trial, comparable interaction of the subjects with the instructor is desirable. Fourth, sampling was not preceded by a power estimation; instead participants that were available were included. Fifth, blinding was not possible. Sixth, co-intervention effects cannot be ruled out. Therefore, larger and longer term full-scale trials with a randomised protocol are required for the future.

As SK&P appears to have the potential to generate positive effects on wellness, it would be interesting to conduct studies that also focus on physiological parameters, such as biochemical markers, as this can give indications as to the mechanism that underlie the effect of these practices, as well as providing a means to objectively test the efficacy of this program. It would also be interesting to conduct additional qualitative studies that specifically focus on psychological experiences during SK&P as this appears to be a method with the potential of generating insight into the psyche.

The need for well-documented methods for increasing wellness is large. There exist a number of techniques, which could increase quality of life and health for many people but they may have shortcomings by different criteria. Data presented here suggest that SK&P may be an efficient and practical method to increase wellness and thus further evaluations are warranted.

## Conclusion

The current findings suggest that adult participants of normal health can improve their wellness by learning and applying a program based on yoga and yogic breathing exercises. This pilot study, with its limitations discussed above, has allowed a number of recommendations to be made to facilitate the design of a large-scale trial. These easily learned and applied cost-effective yogic practices can thus be offered as an intervention to the adult population at large to relieve psychosocial stress and its associated disorders; this in turn may result in the prevention of a score of mental and physical disorders. Full-scale trials are required to conclusively assess the validity of this hypothesis.

## Competing interests

The author(s) declare that they have no competing interests.

## Authors' contributions

AK carried out the design of the study, supervision of data collection, and in data analysis, as well as drafting and finalizing of the manuscript. SB assisted with data collection and analysis. KA helped design the study, was the major person in data collection, and drafted the manuscript. TN was the main person in analysing the data and performed the statistical analysis. FS participated in the design and coordination of the study as well as in drafting and finalizing of the manuscript. All authors read and approved the final manuscript.

## Pre-publication history

The pre-publication history for this paper can be accessed here:


